# Correlates, determinants, and effectiveness of childcare educators’ practices and behaviours on preschoolers’ physical activity and eating behaviours: a systematic review protocol

**DOI:** 10.1186/s13643-015-0011-9

**Published:** 2015-02-21

**Authors:** Stéphanie Ward, Mathieu Bélanger, Denise Donovan, Amanda Horsman, Natalie Carrier

**Affiliations:** Université de Sherbrooke, Centre de formation médicale du Nouveau-Brunswick, Pavillon J.-Raymond Frenette 18, rue des Aboiteaux, Moncton, E1A 3E9 NB Canada; Université de Moncton, Centre de formation médicale du Nouveau-Brunswick, Pavillon J.-Raymond Frenette 18, rue des Aboiteaux, Moncton, E1A 3E9 NB Canada; Université de Moncton, École des sciences des aliments, de nutrition et d’études familiales, Pavillon Jacqueline-Bouchard 51 Antonine-Maillet Avenue, Moncton, E1A 3E9 NB Canada

**Keywords:** Physical activity, Healthy eating, Childcare educator, Preschool child, Childcare centre

## Abstract

**Background:**

Worldwide, approximately 12% of children under the age of 5 are either overweight or obese. As many young children spend 30 h or more per week in childcare centres with childcare educators. Targeting childcare educators as role models may prove an effective strategy for the promotion of healthy eating and physical activity. This manuscript describes the methods to systematically review existing literature relating to how childcare educators influence children’s healthy eating and physical activity behaviours, as well as the links between specific practices and behaviours of childcare educators and children’s healthy lifestyle behaviours.

**Methods:**

Relevant peer-reviewed studies will be identified through a computerized literature search in six databases: PubMed, The Cochrane Library, Science Direct, CINAHL, Wiley and SportDiscus. Quantitative studies written in English or French reporting the correlates, predictors or effectiveness of childcare educators’ practices and behaviours on preschoolers’ healthy eating and physical activity behaviours will be included. The quality of retained studies will be assessed using the Quality Assessment Tool for Quantitative Studies. Descriptive summary statistics of study characteristics will be reported as well as the study designs and exposure and outcome measures. Inter-rater agreements for study selection and quality assessments will be reported and unadjusted, and adjusted results will be presented. Reporting of the systematic review will follow the Preferred Reporting Items for Systematic Reviews and Meta-Analyses (PRISMA) guidelines.

**Discussion:**

This systematic review will contribute to a better understanding of the potential of childcare educators as role models for young children, as well as the influence (or impact) of their behaviours and intervention on children’s short- and long-term health. It will provide important information that could be used to improve obesity prevention strategies and initiatives, as well as to guide the improvement or implementation of effective healthy eating and physical activity policies in childcare centres.

**Systematic review registration:**

PROSPERO (CRD42014012973)

## Background

Childhood obesity has been recognized as one of the greatest public health challenges of the 21st century [1]. Between 1990 and 2010, the worldwide prevalence of overweight and obesity in children under 5 years of age has increased from 4.2% to 6.7% [2]. In 2010, it was estimated that 43 million children were either overweight or obese and this number is likely to rise to 60 million by 2020 [2]. Moreover, an estimated 92 million children were at risk of being overweight because of changing patterns in nutrition and physical activity [2]. In developed countries, the prevalence of overweight and obesity affects more than 8 million 0 to 5 year-olds which is nearly double the prevalence observed in developing countries [2]. The prevalence of childhood overweight and obesity in developed countries is expected to rise from 11.7% in 2010 to 14.1% by 2020 [2].

Children with an elevated body mass index have a greater risk of developing chronic diseases, such as cardiovascular diseases, hypertension and type 2 diabetes in adulthood [3-5], as well as developing these diseases earlier in life [6]. Some consequences of obesity can also be seen in childhood [7]. Over half of children between the ages of 5 and 10 who are overweight have at least one risk factor for cardiovascular diseases, such as hypertension, hyperlipidemia or elevated insulin levels [8]. Children who are overweight are also at risk for emotional distress and compromised social well-being [9]. Furthermore, children who become obese before the age of 6 have an increased risk of being obese later in childhood [10] and are at least four times more likely to become obese adults [11,12].

Although the origins of obesity are complex, childhood obesity is primarily the result of an imbalance between energy intake and energy expenditure [13]. Many environmental factors, found in various settings such as in the home or in schools and neighborhoods, promote the consumption of unhealthy foods and reduce physical activity in children [13]. Preschoolers depend primarily on adults to offer healthy diet and physical activity-related opportunities. Therefore, adults have a large influence on children’s lifestyle patterns. While parents are the primary caregivers, a significant percentage of preschoolers receive formal childcare outside the home. In 2010, 80.6% of children between the ages of 3 and 5 living in developed countries received formal childcare [14]. Among the European countries, childcare attendance is particularly high, with almost universal coverage in countries such as Belgium, France and Spain [14]. In other developed countries, attendance rates vary from 47.3% in Canada, 66.5% in the United States and up to 90.3% in Japan [14]. Hence, it has been suggested that childcare settings could be key locations for promoting healthy eating and physical activity behaviours in young children [15] and could have an impact on their weight throughout childhood [16].

A recent systematic review reported the impact of various types of healthy eating interventions delivered in childcare centres on children’s food choices [17]. The review included single intervention studies which involved the modification of vegetable servings, and educational interventions which were delivered either by teachers, trained individuals or nutritional educators. Multicomponent interventions were also reviewed and included a variety of nutritional-related activities and environmental changes such as implementing healthy school policies and increasing the availability of fruit and vegetables [17]. Results indicated that healthy eating interventions in childcare centres can positively influence children’s consumption of vegetables and fruit as well as improving their nutrition-related knowledge [17]. Another recent systematic review of physical activity interventions in childcare centres found that physical activity could be promoted by limiting the number of children playing at one time, using ground markings and equipment, and focusing on goal-setting or reinforcement [18]. Therefore, intervention studies have shown that childcare centres are settings that can offer a variety of strategies and opportunities to help children adopt healthy lifestyle patterns. Despite these recent literature reviews, it remains unclear how childcare educators’ practices or behaviours influence, predict or correlate with children’s physical activity and healthy eating behaviours.

Bandura’s theory of observational learning suggests that children’s behaviours can be influenced by the people they observe in their environment [19]. For example, a study found that parents who role-modelled healthy lifestyles influenced preschoolers’ consumption of vegetables, fruit and junk food and level of sedentary behaviour [20]. Since children often spend close to, or more than 30 h a week in childcare centres, [21,22] many of them spend nearly as much of their waking hours with their childcare educators as with their parents. This suggests that childcare educators may also have a significant influence on young children’s eating behaviours [23] and physical activity behaviours [16].

### Objectives

This review will provide a summary of empirical studies that have examined the influence of childcare educators on children’s eating behaviours and physical activity in preschoolers. Specifically, this systematic review will identify how childcare educators’ practices and behaviours predict or are correlated with preschoolers’ physical activity and eating behaviours in childcare centres and assess the effectiveness of interventions involving a modification of educators’ practices and behaviours on children’s own behaviours. The main goal of this review will be to identify the potential role of childcare educators as models for the development of healthy eating and physical activity behaviours of children, as well as to suggest avenues for future research.

## Methods/design

### Design

This systematic review will follow the guidelines of the Preferred Reporting Items for Systematic Reviews and Meta-Analyses (PRISMA) statement [24]. The PRISMA statement consists of a 27-item checklist that ensures the transparency and complete reporting of systematic reviews.

### Eligibility criteria

In order to be included in this review, studies will need to 1) assess the unique contribution of childcare educators’ practices or behaviours on children’s eating or physical activity behaviours, 2) focus on physical activity, healthy eating or both outcomes of preschoolers attending childcare centres and 3) be published in either English or French. Studies for which secondary outcomes and analyses meet the above criteria will also be included in this review. Studies of multi-component interventions will be excluded if it is not possible to discern whether the effects reported relate to a variable representing childcare educators’ practices/behaviours. Consistent with the Theory of Observational Learning, studies will also be excluded if the educator-level variable of interest does not relate to practices or behaviours they model.

#### Types of studies

In this systematic review, quantitative studies found in peer-reviewed journals will be included. In order to assess the effectiveness of interventions involving the modification of educator’s practices and behaviours on children’s own behaviours, the review will include randomized controlled trials (RCT), quasi-randomized, cluster-randomized trials and non-randomized controlled trials. Cross-sectional studies will be included to assess the correlates of educators’ practices and behaviours and children’s eating and physical activity behaviours. Finally, in order to determine how childcare educators’ practices and behaviours predict children’s behaviours, cohort (prospective and retrospective) and case-controlled studies will be included. Although the inclusion of non-experimental studies in systematic reviews is debated [25-27], the value of including data from these types of studies in reviews of health interventions is increasingly recognized [26,28]. Specifically, if search results indicate a limited number of experimental or interventional studies focusing specifically on childcare educators, the use of non-randomized or observational-type evidence can provide the justification for future RCTs [25].

Although systematic reviews and meta-analyses of healthy eating physical activity or both interventions will not be included in the review, the reference lists will be checked to ensure that all potentially relevant studies have been identified. Since this review aims to assess the influence of childcare educators on children’s nutrition and physical activity behaviours, qualitative studies will not be included. Other research articles that will not be considered for this review include opinion articles, government or organizational reports, systematic reviews or literature reviews of obesity interventions, books or book chapters, conference abstracts or proceedings, dissertations, theses, blogs, editorials and newsletters.

#### Population of interest

This review will focus on preschoolers attending childcare centres. A childcare centre is a facility where care is provided for infants, toddlers and/or preschoolers by a person other than the child’s legal guardian [29]. These may also be referred to as kindergartens, nurseries or preschools in different countries and may be located in homes or institutional settings, all of which will be considered in this review.

The preschool years relate to a stage of development where preschoolers increase their independence, experience broader social circumstances and expand their ability to control behaviour [30]. It is at this stage that children begin to interact with a larger circle of adults and peers [30], therefore making preschoolers particularly susceptible to external social influences. Preschoolers are generally characterized as children between the ages of 3 and 5 who have not yet attended school [30]. However, PubMed defines preschoolers as children who are between the ages of 2 and 5. Therefore, this definition will be used in this review. In cases where studies have included children of less than 2 or more than 5 years of age, those for which separate analyses were computed for children in our target age group will be included. In the latter case, only data from the age range of interest will be reported.

To be included in the review, studies will need to include a measure of childcare educators’ specific practices or behaviours. A childcare educator will be defined as a person employed by a childcare centre who instructs children or provides care for children. There will be no restrictions as to the gender, ethnicity or socioeconomic statuses of any of the participants.

#### Outcome measures

Objectively and subjectively measured physical activity and eating behaviours of preschoolers will be the primary outcomes of this review. Physical activity-related outcomes can include intensity levels, duration of physical activity, frequency of physical activity or sedentary behaviour (e.g. screen time). Eating behaviours can include types of food eaten (e.g. vegetables, fruits, high-fat foods), nutrient intake (e.g. calcium, saturated fat), meal-time behaviours and attitudes (e.g. openness to try new foods) or nutrition-related knowledge.

### Search methods

The search strategy, including keywords and choice of database selection, will be developed in collaboration with an experienced medical research librarian (AH). A computerized literature search will be conducted in PubMed, The Cochrane Library, Science Direct, CINAHL, Wiley and SportDiscus. Specific search strategies will be initially formulated in PubMed and adapted for each database (Table 1). The reference lists of identified articles will also be reviewed to ensure that all relevant studies are retrieved. All references emerging from the selected search strategies will be exported into the reference manager, Mendeley®, where duplicates will be removed.

**Table 1:** **Search strategy developed in PubMed**

**Database**	**Search strategy**
PubMed (MEDLINE)	((“Motor Activity” [Mesh:noexp] OR “motor activity” [All Fields] OR “Exercise” [Mesh] OR “exercise” [All Fields] OR “Play and Playthings” [Mesh] OR “active play” [All Fields] OR “play” [All Fields] OR “Accelerometry” [Mesh] OR “Accelerometer” OR “Physical Exertion” [Mesh] OR “Physical Exertion” [All Fields] OR “Physical Activity” [All Fields] OR “Movement” [Mesh:noexp] OR “physical activity intensity” [All Fields]) OR (“Food Preferences” [Mesh] OR “Food Preferences” [All Fields] OR “Food Behaviours” [Mesh] OR “Food Behaviours” [All Fields] OR “feeding behaviour” [MESH] OR “eating behaviour” [All Fields] OR “child nutrition sciences” [MESH] OR “child nutrition sciences” [All Fields]) OR (“Obesity/prevention and control” [Mesh] OR “obesity” [All Fields] OR “obesity prevention” [All Fields])) AND (“Caregivers” [Mesh] OR “Caregivers/psychology” [MESH] OR “Caregivers” [All Fields] OR “Faculty” [Mesh] OR “educator” [All Fields] OR “childcare provider” [All Fields] OR “childcare worker” [All Fields]) AND (“Schools, Nursery” [Mesh] OR “nursery schools” [All Fields] OR “Childcare” [Mesh] OR “Child Day Care Centers” [Mesh] OR “Child Day Care Centers” [All Fields] OR “Daycare” [All Fields] OR “preschool” [All Fields] OR “child, preschool” [MeSH Terms]) NOT (“infant” [MeSH Terms] OR “infant” [All Fields])

### Selection of studies

Titles and abstracts of studies emerging from the searches will be checked independently by two investigators (SW and MB), who will then check each other’s references [27]. In the case of a disagreement between the two investigators, the full-text will be checked. The first author (SW) will review the full-text of all the studies and will assess them against the inclusion criteria. A second review of all the eligible or potentially eligible studies will be independently assessed by one of three other investigators (MB, DD, NC), each reviewing one-third of the articles. Studies that do not meet the inclusion criteria or for which full-text versions could not be found or obtained, will be excluded. Disagreements between the reviewers as to the relevance of the study will be resolved through discussion among all four authors. A flow diagram of study selection will be constructed along with the reasons for inclusion and exclusion of the studies, as recommended in the guidelines for reporting systematic reviews presented in the PRISMA statement [24]. The full-text of all studies meeting the inclusion criteria will be imported and stored in Mendeley®.

### Data extraction and management

Data will be entered into an electronic study-specific data extraction sheet by four independent investigators. The first author will initially extract data from all articles, while the second, third and fifth authors will each, independently, extract data from one-third of all included publications. Disagreements and missing data problems will be resolved through discussion among all the authors. Extracted variables will include 1) study characteristics (e.g. author names, year, title of the study, country of origin), 2) type of study, 3) sample characteristics (size, age range or mean age of participants, gender of participants, socio-demographic characteristics), 4) aim and description of the study or intervention, 5) methods of assessment or tools and 6) key outcomes and data.

### Quality assessment

Each eligible study will be assessed for quality using the validated Quality Assessment Tool for Quantitative Studies, developed by the Effective Public Health Practice Project (EPHPP) [31]. This tool was developed in order to provide high quality systematic reviews of articles relating to public health topics (31). Eight aspects of quality are assessed: 1) selection bias, 2) study design, 3) confounders, 4) blinding, 5) data collection methods, 6) withdrawals and dropouts, 7) intervention integrity and 8) analysis, leading to an overall methodological rating of strong, moderate or weak [31]. The quality of all the included studies will be assessed by the first author. The second, third and fifth authors will each check one-third of the publications for completeness and accuracy of the quality assessment. Differences in the quality assessment will be resolved by discussion among all of the authors.

### Data analysis and reporting of findings

Data synthesis will begin by presenting a descriptive summary of the included studies’ characteristics. Summary tables describing the studies and their methodological quality assessment will also be provided. The relevant outcomes of this review (healthy eating and physical activity) will be reported and discussed separately. Percentage of agreement between authors as to study inclusion and quality assessment will be reported, as well as the kappa estimates and *P* values.

As recommended by the Cochrane Collaboration, randomized trials and non-randomized studies will be presented separately [27]. For the purpose of this review, types of studies will be separated to reflect those assessing the effectiveness, predictors and correlates of childcare educators’ practices or behaviours on children’s eating and physical activity behaviours. Whenever possible, for studies which assess effectiveness of interventions, pooled estimates of between group differences will be provided; for studies related to predictors, childcare estimated longitudinal effects will be reported by providing pooled estimates of within and between group differences in change, odd ratios or risk ratios; and pooled correlations or odd ratios will be computed for results from cross-sectional studies. Nevertheless, it is anticipated that there will be a large heterogeneity of outcomes, methods and measurement tools. Therefore, data will be narratively synthesized for each individual study and both adjusted and non-adjusted results will be reported.

The strengths and limitations of each of the included studies will also be reported. Finally, recommendations for future research possibilities and potential implications for obesity prevention and public health will be discussed.

## Discussion

### Implications for health and health research

Overweight and obesity rates continue to raise health and economic concerns around the globe. Obesity prevention interventions targeting children are necessary in order to prevent the onset of chronic diseases later in life. Focusing on promoting healthier eating and physical activity behaviours in childcare centres could be an effective way of reducing overweight and obesity in young children, and helping them adopt healthier lifestyle patterns that could be maintained throughout childhood, adolescence and adulthood.

Given the importance of adults’ role in modelling, teaching, and encouraging behaviours, childcare educators could have the potential to influence the adoption of physical activity and healthy eating behaviour of children in their care. This systematic review will provide important information for childcare educators, directors, parents, researchers and decision-makers, who are looking for ways of preventing obesity and encouraging healthy lifestyle patterns.

This review is expected to provide a foundation for ongoing and new research to develop effective interventions and resources for childcare centres. One potential by-product of this review is the integration of nutrition and physical activity concepts in childcare educators’ academic curriculum and the development of continued education, professional development courses or webinars for childcare educators.

### Strengths and limitations of the review

This systematic review will be the first to review studies on childcare educators as role models for healthy lifestyle behaviours in preschoolers. Strengths of this review include the detailed systematic approach for searching articles, the use of a validated tool aimed at assessing quality of various types of studies relating to public health, not restricting the publication period and including studies published in both English and French. Limitations of this review must also be acknowledged. Among them, is the risk of integrating reporting bias since the review will involve the judgments of the authors. However, this risk of bias will be reduced by the independent reviews of each paper by three investigators. This study will only include quantitative studies, which will limit findings from qualitative studies that may provide insight on why certain practices and behaviours of childcare educators are observed or not in the centres. Finally, we will not be reporting the impact of childcare educators on weight or anthropometric characteristics of children.

### Dissemination

The findings from this systematic review will be disseminated for scientific peer-reviewed publications and will be the subject of conference presentations. This review will also be disseminated to health and education researchers and to policy makers and stakeholders working for government and non-profit organizations related to education and health promotion. The ultimate goal of this review is to produce key information that will be used to improve obesity prevention strategies and initiatives, as well as to provide guidance for improving or implementing effective healthy eating and physical activity policies in childcare centres. This review will provide insight on the extent to which childcare educators may be important role models for children’s healthy lifestyle behaviours, and how their involvement in healthy eating and physical activity promotion may contribute to healthier children.

## Abbreviations

EPHPP: Effective Public Health Practice Project
PRISMA: Preferred Reporting Items for Systematic Reviews and Meta-Analyses
RCT: randomized controlled trials
